# Size-dependent *foraging *gene expression and behavioral caste differentiation in *Bombus ignitus*

**DOI:** 10.1186/1756-0500-2-184

**Published:** 2009-09-16

**Authors:** Yosuke Kodaira, Hajime Ohtsuki, Jun Yokoyama, Masakado Kawata

**Affiliations:** 1 Department of Ecology and Evolutionary Biology, Graduate School of Life Sciences, Tohoku University, Sendai, Miyagi 980-8578, Japan; 2 Department of Biology, Faculty of Science, Yamagata University, Yamagata-shi, Yamagata 990-8560, Japan

## Abstract

**Background:**

In eusocial hymenopteran insects, *foraging *genes, members of the cGMP-dependent protein kinase family, are considered to contribute to division of labor through behavioral caste differentiation. However, the relationship between *foraging *gene expression and behavioral caste in honeybees is opposite to that observed in ants and wasps. In the previously examined eusocial Hymenoptera, workers behave as foragers or nurses depending on age. We reasoned that examination of a different system of behavioral caste determination might provide new insights into the relationship between *foraging *genes and division of labor, and accordingly focused on bumblebees, which exhibit size-dependent behavioral caste differentiation. We characterized a *foraging *gene (*Bifor*) in bumblebees (*Bombus ignitus*) and examined the relationship between *Bifor *expression and size-dependent behavioral caste differentiation.

**Findings:**

A putative open reading frame of the *Bifor *gene was 2004 bp in length. It encoded 668 aa residues and showed high identity to orthologous genes in other hymenopterans (85.3-99.0%). As in ants and wasps, *Bifor *expression levels were higher in nurses than in foragers. *Bifor *expression was negatively correlated with individual body size even within the same behavioral castes (regression coefficient = -0.376, P < 0.001, all individuals; -0.379, *P *= 0.018, within foragers).

**Conclusion:**

These findings indicate that *Bifor *expression is size dependent and support the idea that *Bifor *expression levels are related to behavioral caste differentiation in *B. ignitus*. Thus, the relationship between *foraging *gene expression and behavioral caste differentiation found in ants and wasps was identified in a different system of labor determination.

## Background

Animal foraging behavior is a particularly interesting phenomenon from both ecological and evolutionary perspectives [1,2]. Although understanding the genetic basis of individual foraging behavior is important for evolutionary studies, in most case the genes controlling this behavior have not been identified. Candidate genes have, nevertheless, been identified in insects, and genetic analyses of behavioral differences have been conducted. For example, a *foraging *gene in the fruit fly (*Drosophila melanogaster*) differentiates behavior via a two-allele system: individuals with an R allele (rovers) show higher activity for food materials than those with a for allele (sitters; [3]). The same locus is also related to sucrose response, resistance to high temperature, and learning [4-6].

*Foraging *genes, members of the cGMP-dependent protein kinase (PKG) family, are also found in social insects and are related to the division of labor through behavioral caste differentiation in colonies. In honeybees (*Apis mellifera*), *foraging *gene (*Amfor*) activity differs according to worker age, with gene expression levels increasing with increasing age. Young workers with low expression levels (~ 3 weeks from emergence) act as nurses, whereas older workers with high expression levels (≥3 weeks from emergence) act as foragers [7]. Experimental administration of cGMP has been demonstrated to induce a change from nurses to foragers [8]. In contrast, other findings indicate opposite patterns of *foraging *gene expression among different behavioral castes. Although age-dependent division of labor has also been observed among other social hymenopteran insects, the expression levels of *foraging *genes were found to be higher in nurses than in foragers in two known instances: the foraging ant (*Pogonomyrmex barbatus*) and the common wasp (*Vespula vulgaris*; [9,10]). Furthermore, in the ant (*Pheidole pallidula*), larger individuals with higher PKG activity are predisposed to defend the nest (defenders), whereas smaller individuals are the foragers with lower PKG activity [11]. Lucas and Sokolowski [11] further demonstrated that the pharmacological activation of PKG increased defense and reduced foraging behavior. This study confirmed that lower expression levels produce foragers even in size-dependent behavioral caste differentiation, whereas higher expression produces defenders rather than nurses. Thus, investigation of other social insects with size-dependent nurse/forager behavioral changes might provide additional supportive evidence for the control of behavioral castes by *foraging *genes.

Bumblebees (*Bombus *spp.), which belong to the same family as honeybees, are also social insects but show a different pattern of division of labor. Bumblebees exhibit a unique form of collective nursing, placing several larvae into a single brood chamber and serving food as a lump. Such nursing consequently induces considerable variation in worker size. Worker size is accordingly the major determinant in behavioral castes of bumblebees, although age is also involved to a certain degree. In general, large workers act as foragers and small workers perform as nurses [12,13]. If patterns of gene expression similar to those observed in ants and wasps were to be observed in the size-dependent behavioral caste differentiation system in bumblebees, the relationships between higher (or lower) expression of *foraging *genes and nurses (or foragers) would be clearer.

We predicted that expression of the *foraging *gene would be size dependent since there is a size-dependent behavioral caste differentiation system in bumblebees. Thus, in this study, we characterized the *foraging *gene (*Bifor*) of the Japanese bumblebee (*Bombus ignitus*) and examined its expression patterns in workers. The purpose of this study was to examine the relationship between the *foraging *gene expression level and nurse-forager size difference and to compare the findings with those of previous studies.

## Methods

*Bombus ignitus *is distributed in Japan (Honshu, Shikoku, Kyushu), the Korean Peninsula, and China [14]. Adults (workers) collected in the wild (Sendai, Miyagi Pref., Japan) and those from commercially reared colonies (Agrisect) were used to determine the nucleotide sequences of *foraging *and *ribosomal protein 49 *(*Rp49*) genes. Measurements of body size and mRNA expression analyses were conducted using the commercially reared colonies.

Identification of behavioral castes was achieved by conducting behavioral observations in a colony within an experimental enclosure inside a room (see Additional file 1 for the detailed description for the experimental enclosure and the identification of individual bees). Observations and collection of workers were conducted for two colonies (nests 1 and 2). Nest 1 was observed from September 19 to October 1, 2008, and nest 2 was observed from October 22 to November 3, 2008. In commercially reared colonies, reproductive individuals breed throughout the year; hence, worker individuals can behave as foragers or nurses. The division of labor in such colonies is normally observed in October and November, as is observed in wild colonies during their breeding season.

The observations were made between 7:00 and 10:00, and the bees were individually returned to their colonies until 18:00. After this time, all the workers present outside the colonies were collected and returned to the colonies. Having numbered the bees, they were allowed to independently choose to behave as foragers or nurses for 3 days. Although the bees were placed in relatively small artificial environments, important components required for their foraging behavior (e.g., a sufficient spatial scale for recognition of the direction of food sites and the distance between the nest and food) were provided within the enclosure. We therefore assume that the bees exhibited a normal foraging behavior.

By observing the workers for 3 days or more after numbering, behavioral castes were identified as follows. Workers making three or more trips to collect food within a 3-h observation period were identified as "foragers." Those not making foraging trips during the observation period were defined as "nurses." Foragers were collected at two time periods: while bees visited flowers in the daytime (active foragers) and while they rested in the colony at night (resting foragers). In the daytime, foragers were caught while they were perched on flowers or while they were flying. Nursing behavior could also be observed at night; thus, nurses were collected at night. All the collected bees were immediately fixed in liquid nitrogen and stored at -80°C until use. In this study, head width was used as an indicator of body size.

The expression levels of the *foraging *gene (*Bifor*) were determined relative to the expression levels of *Rp49*. The detailed methods used to determine the nucleotide sequence of *Bifor *and for real-time PCR analyses are described in Additional file 1.

## Results

The full length of the coding region of *Bifor *was determined as 2004 bp (668 aa). On comparing with the functional region identified in *Amfor *(two regions in CAP_ED, S_TKc, S_TK_X; Ben-Shahar et al., 2002), *Bifor *showed high identities with other hymenopteran *foraging *genes (477 aa: *A. mellifera*, 99.0%; *Nasonia vitripennis*, 95.0%; *V. vulgaris*, 85.3%).

**Figure 1:**
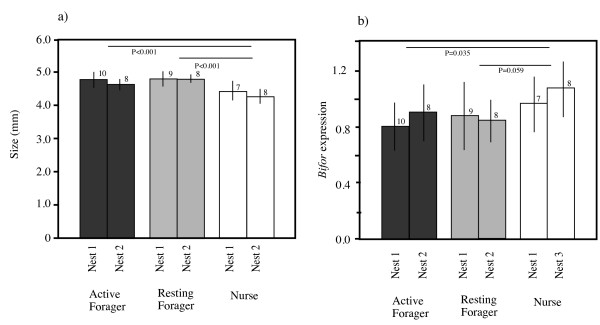
**(a) Head width (as an indicator of body size) and (b) *Bifor *expression levels of active foragers, resting foragers, and nurses**. Figures above the bars indicate the number of individuals examined. Vertical lines indicate standard deviation.

**Figure 2:**
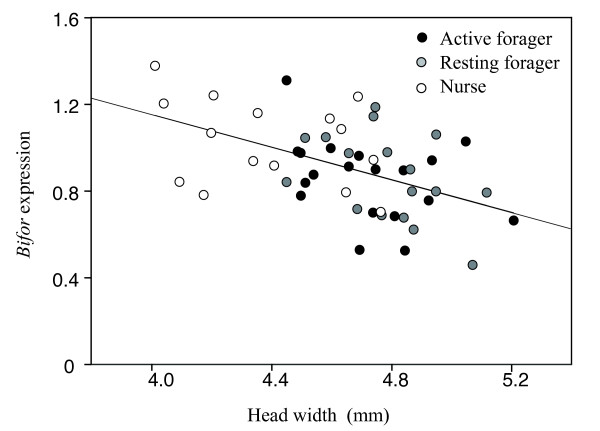
**The relationship between head width (as an indicator of body size) and *Bifor *expression level for the different types of workers**.

There was a significant difference in head width among foragers (active and resting) and nurses (2-way ANOVA: F_2,47 _= 15.25, *P *< 0.0001; Figure 1a), but not among the size of the nests (F_1,47 _= 2.324, *P *= 0.1341). The head width was significantly wider in the foragers (both active and resting) than in the nurses (Tukey's HSD test: active vs. resting foragers, *P *= 0.604; active foragers vs. nurses, *P *< 0.001; resting foragers vs. nurses, *P *< 0.001). Similarly, there was a significant difference in the level of *Bifor *expression among active and resting foragers and nurses (2-way ANOVA, F_2,47 _= 3.931, *P *< 0.026; Figure 1b), but not among the size of the nests (F_1,47 _= 1.322, *P *= 0.256). Nurses exhibited a significantly higher level of *Bifor *expression (relative to the expression level of *Rp49*) than active foragers (Tukey's HSD test: *P *= 0.035) and a nearly significantly higher expression than resting foragers (*P *= 0.0594). A regression analysis of the combined data for all the workers showed that there was a significant negative relationship between *Bifor *gene expression level and head width [regression coefficient, -0.376 (SE, 0.09), *P *< 0.001; Figure 2].

## Discussion

As found in *B. terrestris *[13], size-dependent behavioral caste differentiation was confirmed in *B. ignitus *in this study. Bumblebees also exhibit certain aspects of division of labor according to worker age: young bees remain in colonies, whereas older bees tend to become foragers [15]. However, all young bees act as nurses in the first few days after emergence, and thereafter large-sized bees are likely to switch their functions to foragers, whereas small-sized bees remain nurses [12,16]. Therefore, except for the first few days after emergence, the behavioral caste of bumblebees is determined by worker size. As identification of behavioral caste was conducted at least 3 days after the numbering in this study, we could only demonstrate the size-dependent determination of behavioral castes in bumblebees.

*Bifor *expression levels were higher in nurses than in foragers, and there was a clear negative relationship between body size and *Bifor *expression level even within the same behavioral castes (within the foragers, regression coefficient, -0.379 [SE, 0.02], *P *= 0.018; within the nurse, regression coefficient, -0.251 [SE, 0.19], *P *= 0.224). Although there was no significant relationship between the expression levels of *Rp49 *and body size, it is possible that a somewhat weak positive correlation between *Rp49 *expression levels and body size affected the negative relationship between body size and relative *Bifor *expression level. However, multiple regression analysis showed that the absolute expression level of *Bifor *(values not relative to *Rp49 *expression) was significantly negatively related to size (partial regression coefficient with size, -1.2632, *P *= 0.0022) and positively related to *Rp49 *expression (partial regression coefficient with *Rp49 *= 0.466, *P *< 0.0001). These findings indicated that *Bifor *expression levels are negatively correlated to body size irrespective of *Rp49 *expression levels.

The results of our study indicate that both *Bifor *expression and behavioral caste differentiation is highly size dependent. They also suggest that in species with size-dependent behavioral caste differentiation, foragers exhibit lower expression of the *foraging *gene than nurses. This pattern has also been observed in species exhibiting age-dependent division of labor such as *P. barbatus *and *V. vulgaris *[9,10], and in size-dependent forager-defender behavioral caste differentiation in *Pheidole pallidula *[11]. These findings support the hypothesis that a lower expression level of *Bifor *is associated with foraging behavior and a higher expression level with nursing behavior. A negative relationship between body size and *Bifor *expression level was observed even within the same labor group. Thus, it can be speculated that *Bifor *expression might be suppressed with increasing body size, and that there might be a threshold level, below which individuals become foragers. However, further experiments, such as cGMP administration, will be required in order to confirm the relationships among behavioral caste, body size, and *foraging *gene.

We also found that there was little or no difference in *Bifor *expression between resting and active foragers, indicating that *Bifor *expression does not directly affect the foraging activity of bees but that it may be related to the characteristics required for foraging or nursing. Foragers, but not nurses, have strong circadian rhythms [17], which may affect the regulation of many genes. Thus, the foraging behavior observed during the day might have been caused by clock regulation. However, flying behavior is triggered even in the presence of a small amount of light, whereas foraging behavior is adversely affected by light [18]. Furthermore, sensitivity to light depends on body size [18]. Thus, it is possible that *Bifor *expression might be related to a threshold body size, above which individuals begin to forage in response to light.

## Conclusion

*B. ignitus *exhibits size-dependent behavioral caste differentiation. *Bifor *expression levels were higher in nurses than in foragers, and there was a clear negative relationship between body size and *Bifor *expression level even within the same labor group. These findings indicate that *Bifor *expression is size dependent and support the idea that its expression levels are related to behavioral caste differentiation. Thus, the relationship between *foraging *gene expression and behavioral caste differentiation previously identified in ants and wasps has been confirmed in another social insect with a different system of labor determination.

## Competing interests

The authors declare that they have no competing interests.

## Authors' contributions

YK carried out the behavioral observations, the determination of gene sequences, and real-time PCR analysis. HO assisted with the real-time PCR analysis. JY provided advice on the behavioral and molecular genetic studies. MK supervised the research and wrote the paper. All authors read and approved the final manuscript.

## Supplementary Material

Additional file 1 **Supplemental material and methods**. The detailed descriptions of the methods for Experimental enclosure and identification of individual bees, the nucleotide sequence determination of Bifor and real-time PCR analyses. Click here for file
