# Supplementary material for: Size-dependent foraging gene expression and behavioral caste differentiation in Bombus ignitus
Source: BMC Res Notes. 2009 Sep 16;2:184. doi: 10.1186/1756-0500-2-184 (PMC2751771; doi:10.1186/1756-0500-2-184)
Supplement: Additional file 1 — Supplemental material and methods. The detailed descriptions of the methods for Experimental enclosure and identification of individual bees, the nucleotide sequence determination of Bifor and real-time PCR analyses. [file 1756-0500-2-184-S1.doc]

**Additional file 1. Supplementary materials and methods**

***Experimental enclosure and identification of individual bees***

A colony was set within an experimental enclosure inside a room [2 m (length) × 1 m (width) × 2 m (height)] covered with a 6-mm mesh and provisioned with cut flowers of food plants (mainly *Trifolium pratensis*). Transparent acrylic tubes [15 cm (length) × 18 mm (caliber)] were connected to two entrances of a commercial colony box to observe individuals. In order to distinguish individual workers within the colony, 6-mm circular stickers bearing numbers were affixed to each bee following low-temperature anesthetization.

***Determination of the nucleotide sequence of the B. ignitus foraging gene***

Total RNA was isolated from the heads of collected adults using Isogen (Nippon Gene, Tokyo, Japan). First-strand cDNA was generated from the total RNA using a GeneRacer kit (Invitrogen). PCR amplifications of *foraging* gene 3‘ and 5‘ cDNAs were performed using TaKaRa Taq (TaKaRa). Amplification of cDNA was performed in a 50-μl mixture containing 2.5 units of TaKaRa Taq, 10 mM of Tris-HCl, 50 mM of KCl, 2.75 mM of MgCl_2_, 0.5 mM of each primer, 0.25 mM of dNTPs, and 5.0 μl of cDNA. The following thermal cycle was used for the amplifications: 94°C for 2 min, followed by 30 cycles of 94°C for 30 s, 60°C for 30 s, and 72°C for 90 s, with a final extension at 72°C for 15 min. The primers used for the amplifications are listed in Table 1. PCR was performed using a PCR Express II thermal cycler (ThermoHybaid, Middlesex, UK).

The PCR products were purified by electrophoretic separation on 1.5% SeaPlaque GTG agarose gel (FMC BioProducts, Rockland, ME). These products were cloned using a Zero Blunt TOPO PCR cloning kit (Invitrogen). Colony PCR was performed with primers T7 and T3 or M13F and M13R, and then the products were purified using the same method as described above.

The DNA sequencing reaction was performed using a BigDye Terminator v3.1 Cycle Sequencing Ready Reaction kit (Applied Biosystems, Foster City, CA). The products of the sequencing reaction were purified by ethanol precipitation. Sequencing was performed using an ABI Prism 3130 genetic analyzer (Applied Biosystems) for two or three independent clones of each PCR product. The sequence was deposited with GenBank (accession number AB491725).

***Real-time PCR***

Total RNA was isolated from the heads of workers using RNAiso (TaKaRa). Contaminated DNA was removed from the total RNA solution using DNase I (RNase-free; TaKaRa). The RNA concentration was measured using GeneQuant 100 (GE Healthcare Biosciences) and adjusted to 50 ng/μl. cDNA was subsequently generated using a PrimeScript RT reagent kit (TaKaRa). Real-time PCR was performed on a 20-μl mixture containing 10 μl of SYBR Premix Ex Taq (TaKaRa), 0.4 mM of each primer, and 2.0 μl of diluted (20-fold) cDNA using a LightCycler 350S (Roche). The following thermal cycle was used for the reaction: 95°C for 10 s, followed by 40 cycles of 95°C for 5 s and 62°C for 20 s. The primers used for the amplifications are listed in Table 1. The *Rp49* gene was used as an internal control. The expression level of the *foraging* gene was determined relative to the expression level of *Rp49*. The relative expression was further normalized on an arbitrary scale, where an individual with near-average relative expression values was assigned a value of 1.00.

Individuals whose head size was less than 4 mm were omitted from the analysis since they showed lower *Rp49* expression levels. After such individuals had been omitted, we detected no significant relationship between head size and *Rp49* expression level (regression analysis: regression coefficient = 0.9477, *P* = 0.268).

**Measurement of head size**

In this study, head width was used as an indicator of body size. Just before total RNA isolation, digital images of the heads were recorded using a light microscope (Olympus SZX12) and a digital camera (Olympus Camedia C-4040 Zoom). Using these images, head width was measured with ImageJ (NIH).

**Statistical analyses**

The relationships between head width and *foraging* gene expression, labor division, or differences between the colonies were analyzed by two-way ANOVA and Tukey’s HSD post hoc test. All statistical analyses were carried out using R v.2.2.0 (R Development Core Team, 2005).

**Table 1.** PCR primers used in this study. All primers except the adapter primers were developed in this study.

| Primers | Sequence (5’-3’) |
| --- | --- |
| Primers for *foraging* gene | |
| Amfor_01_F | GACCGTGAAACGTTCAATCAGCTAATCTC |
| Amfor_01_R | GAGATTAGCTGATTGAACGTTTCACGGTC |
| Amfor_02_F | CAGCAACAACACATTATGTCAGA |
| Amfor_02_R | TCTGACATAATGTGTTGTTGCTG |
| Amfor_03_R | GGGAATTCTATAGCGTCGATTCCTTT |
| Amfor_04_R | CGAACCATTTGTGTTTCTGTATTTC |
| Bfor_05_R | ACTCTTCCGAAACCGCCTACACC |
| Bfor_06_F | GCCGATTACTGGTCCCTTGGTGT |
| Bfor_07_F | GATTGCCAGCTATGGGCCATCGA |
| Bfor_07_R | TCGATGGCCCATAGCTGGCAATC |
| Bifor_08_F | ATGGGCACCCTTCGGGAGCT |
| Bifor_09_R | TTAAAAATCATTATCCCAGCCGGAT |
| Bifor_10_F | ATGGGCACCCTTCG |
| Bifor_11_R | TGGAAGCATTGTCGATCGATGGC |
| Bifor_12_R | AGGCCGGTTCTCATCATAATCGT |
| Bifor-RT-2F* | TGGTGATAGACCGTGAAAC |
| Bifor-RT-2R* | GTAGCCAGTGGTCGAAGAT |
| Primers for *ribosomal protein49* gene | |
| Birp49_01_F | TACAGGCCKACAATYGTKAA |
| Birp49_02_R | GCACGTTCWACAATRGMTTTACGTTT |
| Birp49-F* | GAAGTTCATTCGTCATCAGAG |
| Birp49-R* | TTGTCCCTTAAATCGCCTAC |
| Adapter primers | |
| GeneRacer 5’ Primer | CGACTGGAGCACGAGGACACTGA |
| GeneRacer 5’ Nested Primer | GGACACTGACATGGACTGAAGGAGTA |
| GeneRacer 3’ Primer | GCTGTCAACGATACGCTACGTAACG |
| GeneRacer 3’ Nested Primer | CGCTACGTAACGGCATGACAGTG |
| M13 Forward （-20） Primer | GTAAAACGACGGCCAG |
| M13 Reverse Primer | CAGGAAACAGCTATGAC |

*: Primers used for real-time PCR.
